# Cytotoxic Effects of Zoom^®^ Whitening Product in Human Fibroblasts

**DOI:** 10.3390/ma13071491

**Published:** 2020-03-25

**Authors:** Carlos Miguel Marto, Mafalda Laranjo, Anabela Paula, Ana Sofia Coelho, Ana Margarida Abrantes, João Casalta-Lopes, Ana Cristina Gonçalves, Ana Bela Sarmento-Ribeiro, Manuel Marques Ferreira, António Cabrita, Maria Filomena Botelho, Eunice Carrilho

**Affiliations:** 1 Institute of Experimental Pathology, Faculty of Medicine, University of Coimbra, 3000-548 Coimbra, Portugal; amscabrita@ci.uc.pt; 2 Coimbra Institute for Clinical and Biomedical Research (iCBR), area of Environment, Genetics and Oncobiology (CIMAGO), Faculty of Medicine, University of Coimbra, 3000-548 Coimbra, Portugal; mafaldalaranjo@gmail.com (M.L.); anabelabppaula@sapo.pt (A.P.); anasofiacoelho@gmail.com (A.S.C.); mabrantes@fmed.uc.pt (A.M.A.); acc.goncalves@gmail.com (A.C.G.); absarmento@fmed.uc.pt (A.B.S.-R.); m.mferreira@netcabo.pt (M.M.F.); mfbotelho@fmed.uc.pt (M.F.B.); eunicecarrilho@gmail.com (E.C.); 3 Center for Innovative Biomedicine and Biotechnology (CIBB), University of Coimbra, 3000-548 Coimbra, Portugal; 4 Clinical Academic Center of Coimbra (CACC), 3004-561 Coimbra, Portugal; 5 Institute of Biophysics, Faculty of Medicine, University of Coimbra, 3000-548 Coimbra, Portugal; joao.casalta@gmail.com; 6 Institute of Integrated Clinical Practice, Faculty of Medicine, University of Coimbra, 300-075 Coimbra, Portugal; 7 Radiation Oncology Department, Coimbra University Hospital Center, 3000-548 Coimbra, Portugal; 8 Laboratory of Oncobiology and Hematology (LOH) and Universitary Clinic of Hematology, Faculty of Medicine, University of Coimbra, 3000-548 Coimbra, Portugal; 9 Institute of Endodontics, Faculty of Medicine, University of Coimbra, 300-075 Coimbra, Portugal; 10 Coimbra Chemistry Centre, Faculty of Sciences and Technology, University of Coimbra, 3004-504 Coimbra, Portugal

**Keywords:** tooth whitening, hydrogen peroxide, cytotoxicity, fibroblasts, oxidative stress

## Abstract

Tooth whitening procedures are increasing; however, side effects can occur, such as damage to pulp cells, by the whitening products. This study aims to assess the cellular effects promoted by a whitening product, namely, the oxidative stress fostered by the active agent hydrogen peroxide, with and without photoactivation. Additionally, if cellular recovery occurred, we intended to determine the time point where cells recover from the tooth whitening induced damage. Human fibroblasts were exposed to hydrogen peroxide, Zoom^®^, Zoom^®^ + irradiation, and irradiation alone. The following analysis was performed: metabolic activity evaluation by the MTT assay; cell viability, mitochondrial membrane potential, peroxides production, superoxide radical production, and reduced glutathione expression by flow cytometry. We determined the IC_50_ value for all groups, and a dose-dependent cytotoxic effect was verified. At the times analyzed, hydrogen peroxide groups showed no metabolic activity recovery while a cell recovery was observed after 24 h (Zoom^®^) and 48 h (Zoom^®^ + irradiation). Cell death was seen in hydrogen peroxide and Zoom^®^ + irradiation groups, mainly by apoptosis, and the irradiation had a cytotoxic effect per se. This in vitro study supports that whitening products with moderate hydrogen peroxide (HP) concentration have a temporary effect on cells, allowing a cellular recovery.

## 1. Introduction

The improvement of dental esthetics is one of the main reasons people seek dental treatment. Most patients refer to a dissatisfaction with dental coloration caused by pigments in the dental structure [1]. Tooth whitening procedures are thus increasing in the dental office and at home, with hydrogen peroxide (HP) being the main active compound used, since it is effective, safe if correctly used, and low-cost [2,3]. HP concentration in whitening products can be up to 35% or higher, obtained directly or from a chemical reaction using sodium perborate or carbamide peroxide [4].

Hydrogen peroxide is a strongly oxidizing agent of organic and inorganic compounds through the formation of free radicals, such as reactive oxygen species (ROS), which include the superoxide anion, the hydroxyl radical, singlet oxygen, and hydrogen peroxide itself [1,5]. ROS are highly unstable and promote a redox reaction in the tooth structure, decomposing the long pigment chains into smaller, less pigmented, more diffuse and in some cases water-soluble molecules, whitening the tooth [5,6]. ROS formation from hydrogen peroxide can be improved by light systems, quickening the treatment [7,8].

Because the tooth is a semi-permeable structure, whitening agents applied at the tooth surface can diffuse and reach the dental pulp, promoting damage to dental pulp cells, such as a decrease in cell viability and metabolism, inflammatory response, DNA damage, apoptosis, or even necrosis [9,10]. These cytotoxic effects are directly proportional to the concentration of HP applied and cell damage relates to tooth sensitivity, the most clinically related adverse effect of tooth whitening [11]. Manufacturers try to overcome this issue by adding desensitizing, stabilizing, or antioxidant agents to the formulation of whitening products, thus promoting protection from oxidative stress for dental pulp cells and preventing tooth sensitivity [12]. However, the exact composition of these products is often unknown, and thus the ability of these other components to protect cells from oxidative stress may vary, contributing to different in vitro and in vivo biocompatibility reports [9].

Since oxidative stress induced by hydrogen peroxide is a well-documented effect, we aimed to assess the safety in use and cellular response to a dental whitening product. We wanted to determine if the other components present in a tooth whitening product are capable of protecting cells from the oxidative stress fostered by the active agent hydrogen peroxide, with and without photoactivation. Additionally, if cellular protection occurred, we intended to determine the time point where cells recover from the damage induced by the tooth whitening protocols.

## 2. Materials and Methods

### 2.1. Ethical Approval

This study was approved by the Ethics Commission, Faculty of Medicine, University of Coimbra (approval CE-006/2014 and addendum CE-121/2017).

### 2.2. Cell Culture

Human fibroblasts were used to access whitening product cytotoxicity [13,14]. Cells were obtained according to ethical and legislation protocols [15] and maintained at 37 °C in a humidified atmosphere with 95% air and 5% CO_2_ in a cell incubator (Heraeus HeraCell 150 CO_2_–BridgePath Scientific, MD, USA). The cell line was cultured in adherent conditions, with Dulbecco’s Modified Eagle’s Medium (DMEM)/Ham’s F12 Nutrient Mixture (DMEM F12, Sigma D8900), supplemented with 10% heat-inactivated fetal bovine serum (Gibco 2010-09), 100 μM sodium pyruvate (Gibco 11360), and 1% antibiotic/antimycotic (10,000 units penicillin, 10 mg streptomycin and 25 µg amphotericin B, Sigma A5955).

### 2.3. Cell Treatment

The in vitro cytotoxicity evaluation was performed following the protocol recommendations in ISO 10993-5, using a direct test methodology and a 25% HP whitening product, Zoom^®^ (Discus Dental, Ontario, CA, USA) [14,16]. According to the product safety data sheet, the Zoom^®^ whitening product composition water consists of polyethylene-polypropylene glycol, hydrogen peroxide, glycerin, propylene glycol, potassium nitrate, mentha piperita oil, eugenol, 1-hydroxyethane-1,1-diphosphonic acid, iron, and bis(D-gluconato-O1,O2)-,dihydrate. This whitening product was chosen because it is broadly used worldwide at clinical practice and presents an intermediary HP concentration. For experiments, a cellular suspension of 100,000 cells/mL was platted in a 48 multiwell (CLS3548, Corning Costar Corp^®^, MA, USA) and left overnight to allow cell attachment.

For metabolic activity studies, three experimental groups were created: G1—hydrogen peroxide; G2—Zoom^®^, and G3—Zoom^®^ + irradiation. Additionally, for cell viability and oxidative stress studies, a fourth group was inserted: G4—irradiation only. Irradiation in groups 3 and 4 was performed with the manufacturer’s system light (LED light, Discus Dental, Ontario, CA, USA) at 30 cm from plaque to prevent possible cellular heating caused by the light [17]. The exposure time was 45 min, following the manufacturer’s instructions for clinical application. Administration vehicles were used as controls, sterile water in G1 and G4 and dimethylsulfoxide (DMSO) in G2 and G3 groups were used in the same volume for HP and Zoom^®^ administration [18].

### 2.4. Evaluation of Metabolic Activity

Metabolic activity was determined using the colorimetric MTT (3-(4,5-dimethyl thiazolyl-2)2,5-diphenyltetrazolium bromide) assay [19]. The Zoom^®^ product and a 30% H_2_O_2_ solution (Merck) were sequentially diluted to obtain final solutions ranging from 0.3% to 0.000001% of H_2_O_2_ to both compounds. A 10^6^ cell suspension was platted and submitted to the referred concentrations of Zoom^®^ or HP for 45 min. Metabolic activity evaluation was performed at 0, 24, 48, and 72 h after exposure by adding MTT (0.5 mg/mL, Sigma, St. Louis, MO, USA) in PBS, pH 7.4, in the dark at 37 °C for 4 h. The formazan crystals were solubilized with a 0.04 M solution of hydrochloric acid in isopropanol and absorbance was measured using an SLT-Spectra spectrophotometer [19]. Dose–response curves were obtained with the software OriginPro 8.0 (OriginLab Corporation) by adjusting experimental data to the Boltzmann sigmoidal model, according to the equation: MA = 100/(1 + e^(logχ0 − logC)^ × *p*), where MA represents the metabolic activity, C is the concentration, *p* is the slope of the central region of the sigmoid, and χ0 is the IC_50_. The 95% confidence intervals for the IC50 were obtained from the parameters found by curve fitting (logχ0 and respective standard error).

Cytotoxicity was calculated as a percentage of the metabolic activity inhibition in treated cultures, correlated with control groups which were considered as 100%. This procedure allowed us to determine the half-maximal inhibitory concentration (IC_50_) for each condition, which was used in subsequent experiments.

### 2.5. Flow Cytometry Studies

Cell suspensions with 10^6^ cells were prepared as previously described and flow cytometry was performed immediately after application of the compounds. For this analysis, two concentrations were chosen for each group, the respective IC_50_ and 0.01%.

All evaluations were performed in a 6-parameter, 4-color FACSCalibur flow cytometer (Becton Dickinson, San Jose, CA, USA) equipped with a 15 nW argon laser. The number of events obtained in the software CellQuestTM (Becton Dickinson, San Jose, CA, USA), which corresponds to the number of cells analyzed for each condition, was 104. For the analysis and quantification of the results, the software Paint-a-Gate 3.02 (Becton Dickinson, San Jose, CA, USA) was used.

### 2.6. Cell Viability

Cell viability was evaluated through the annexin-V/propidium iodide (AV/PI) incorporation assay, which allows a distinction between live, early apoptotic, late apoptotic/necrotic, and necrotic cells [18]. Briefly, 10^6^ cells were incubated for 15 min in 100 μL of binding buffer, 1 μL of AV-FICT, and 5 μL of IP (KIT Immunotech, Marseille, France), during 15 min at room temperature and in the dark, according to the kit manufacturer’s instructions, followed by flow cytometry analysis [19].

### 2.7. Mitochondrial Membrane Potential Evaluation

The lipophilic cationic fluorescent molecule, 5,5′,6,6′-tetrachloro-1,1′,3,3′-tetraethylbenzimidazolcarbocyanine iodide (JC-1), which exists in two forms, monomers (M) and aggregates (A), depending on membrane potential, was used. This probe is selectively captured by mitochondria, per its membrane polarization/depolarization state, so when membrane potential is high, JC-1 originates aggregates that emit red fluorescence (590 nm), and when mitochondrial potential decreases or when the membrane becomes depolarized, JC-1 is excluded from mitochondria and remains in the cytoplasm as monomers that emits green fluorescence (529 nm). Thus, an estimate of mitochondrial membrane potential can be provided by the ratio between M/A or green/red fluorescence [19]. The 10^6^ cells were incubated for 15 min, at 37 °C in the dark, with 5 mg/mL of JC-1 (Molecular Probes, Invitrogen^®^, Carlsbad, CA, USA). The cellular suspension was washed with PBS and flow cytometry was performed. Results are expressed as the ratio of the mean fluorescence intensity (MFI) of monomers and aggregates.

### 2.8. Detection of Intracellular Peroxides

Production of intracellular peroxides was performed with non-fluorescent probe 2,7-dichlorodihydrofluorescein diacetate (DCFH2-DA). This liposoluble compound enters cells and accumulates at cytosol and is cleaved by intracellular esterases in 2,7-dichlorodihydrofluorescein (DCFH2), which in the presence of peroxides is oxidated to dichlorodihydrofluorescein (DCF), a green fluorescent compound easily visualized with excitation and emission wavelengths of 504 nm and 529 nm, respectively [18]. Emitted fluorescence and intracellular peroxide concentrations are proportional [20]. Briefly, a cell suspension of 10^6^ cells was incubated for 45 min, at 37 °C in the dark, with 5 μM of DCFH2-DA (Molecular Probes, Invitrogen^®^, Carlsbad, CA, USA). After the cells were washed with PBS, the detection was made by flow cytometry.

### 2.9. Detection of Superoxide Radical Production

Radical quantification was performed with a dihydroethidium (DHE) probe, which crosses cellular membranes and is converted into ethidium by the superoxide radical. Ethidium is a fluorescent red compound that interleaves with DNA, remaining in the cell interior and this reaction is relatively specific to the superoxide radical, with minimum oxidation by other molecules [21].

Concisely, 10^6^ cells were resuspended in 1000 μL of PBS and incubated for 10 min in 5 μM of DHE (Sigma 3729) at 37 °C in the dark. After cell wash with PBS, an excitation wavelength of 620 nm was used in flow cytometry [19].

### 2.10. Reduced Glutathione Expression

Reduced glutathione (GSH) evaluation, an antioxidant defense, was performed with the fluorescent compound mercury orange. This compound reacts with GSH and this reaction product emits red fluorescence when excited at 488 nm [22,23]. A cell suspension of 10^6^ cells was incubated for 15 min at room temperature in the dark with 40 mM of mercury orange (Sigma 83377). After washing the cells with PBS, fluorescence was determined at 485/20 nm excitation and 590/35 nm emission [23].

### 2.11. Statistical Analysis

Analysis of results was performed with the software SPSS^®^ (Statistical Package for Social Sciences) version 20.

For the comparison of the dose–response curves, the IC_50_ value and respective standard error were determined, and comparisons were performed using one-way analysis of variance (ANOVA) followed by post-hoc comparisons using Bonferroni’s method for *p*-value correction. For cell viability, mitochondrial membrane potential evaluation, and oxidative stress, the non-parametric Mann–Whitney test was used. A type-I error of 0.05 was considered for all comparisons (*p* < 0.05).

## 3. Results

### 3.1. Metabolic Activity

Since the MTT assay translates cell metabolic activity, it was used to evaluate the cell function and to determine the half-maximal inhibitory concentration (IC50) for each condition and time.

**Figure 1:**
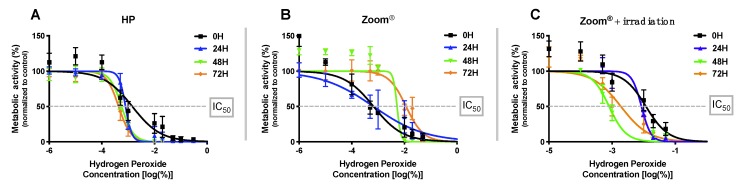
Dose–response curves at 0 h, 24 h, 48 h, and 72 h, for hydrogen peroxide (HP) (**A**), Zoom^®^ (**B**), and Zoom^®^ + irradiation (**C**), expressed as the dose-related inhibition of cell proliferation, determined by the MTT assay. The results express the average of 6 independent experiments ± standard error.

The dose–response curves of the three conditions are shown in Figure 1. As described in detail in the materials and methods section, the metabolic activity values obtained were used to trace adjusted dose–response curves for the different groups and experimental times.

Using the dose–response curves, the half-maximal inhibitory concentration (IC_50_) for each condition was determined, as shown in Table 1, that summarizes the IC_50_ values for the three conditions and times.

Inhibition of metabolic activity promoted by hydrogen peroxide shows no recovery among the analyzed times, even rising from 24 h to 48 h (*p* < 0.001) and from 48 h to 72 h (*p* = 0.003), revealing an increased cytotoxic effect, which is confirmed when comparing 0 h to 72 h (*p* < 0.001).

The whitening agent Zoom^®^ shows higher metabolic activity inhibition at 0 h, and a consistent decrease in cell cytotoxicity can be seen after 24 h. Statistical significance (*p* < 0.001) exists in the comparison between 0 h to 72 h and 24 h to 72 h, evidencing cellular recovery during the analyzed time.

For the combination of Zoom^®^ + irradiation, inhibition of metabolic activity increases until 48 h (*p* < 0.001 for comparison between 0 h and 48 h) and a decrease in metabolic activity inhibition occurs after this time, with *p* = 0.07 for comparison between 48 h and 72 and *p* < 0.001 for 0 h and 72 h.

Paralleling IC_50_ values from HP, Zoom^®^, and Zoom^®^ + irradiation, statistical differences appear between the three groups at 0 h (*p* < 0.001), 24 h (*p* = 0.035), and 72 h (*p* < 0.001), since Zoom^®^ and Zoom^®^ + irradiation present cellular recovery over time, contrary to hydrogen peroxide. Furthermore, at 72 h, a difference between the groups Zoom^®^ and Zoom^®^ + irradiation was found, with *p* < 0.001.

### 3.2. Cell Viability

Because a decrease in the cell metabolic activity was seen, the cell viability was tested to determine if it was associated with cell death.

**Figure 2:**
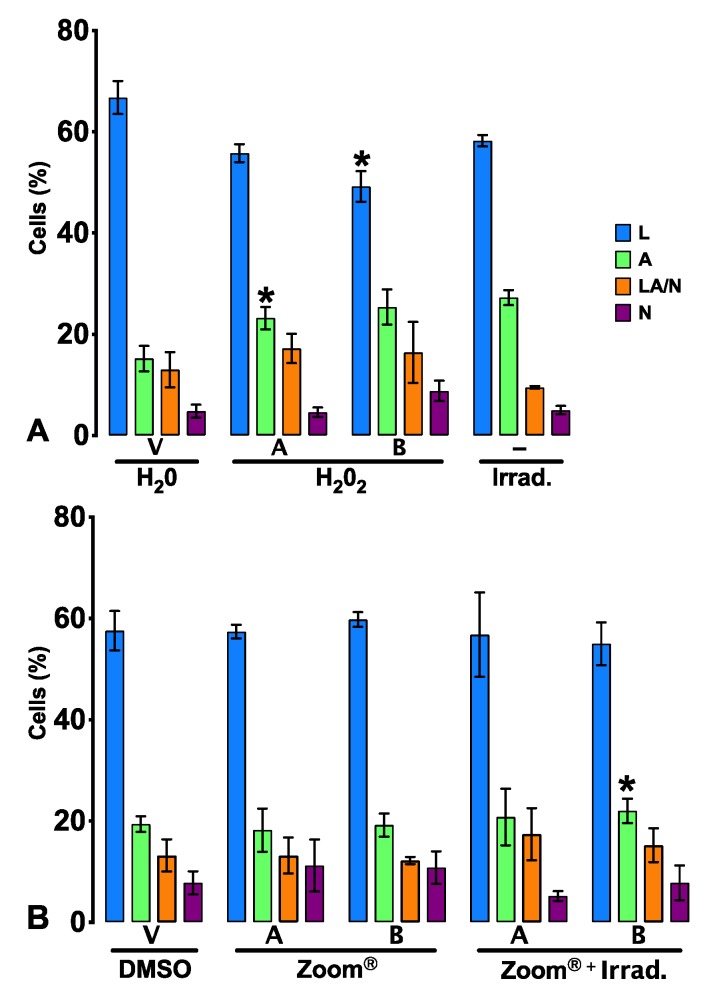
Cell viability determined by flow cytometry using the annexin-V/propidium iodide (AV/PI) incorporation assay. v: vehicle; **–**: no compound administration. Results are presented as the percentage of live cells (V), early apoptosis (A), late apoptosis/necrosis (A/N), and necrosis (N). Statistical differences from the respective control are indicated by *, where * represents *p* < 0.05. The results express the average of 5 independent experiments ± standard error.

Figure 2 shows that all tested conditions induce cell death, mainly by apoptotic events. The percentage of death was significant for all conditions, with a death rate ranging from 40.2% (0.01% Zoom^®^) to 51.8% (IC_50_ HP). The application of IC_50_ HP brings about a significant increase in apoptotic cells (*p* = 0.027), and at 0.01% HP a decrease of live cells occurred (*p* = 0.016) and a tendency towards apoptosis increase was observed (*p* = 0.059).

A significant result is found in group 4, where irradiation only promotes an increase in apoptotic cells (*p* = 0.036).

### 3.3. Mitochondrial Membrane Potential Evaluation

HP is a well-known oxidation agent, and cell exposure to it can trigger apoptosis through the mitochondrial pathway. A hallmark of this process is the loss of mitochondria membrane potential which was evaluated using a specific probe.

**Figure 3:**
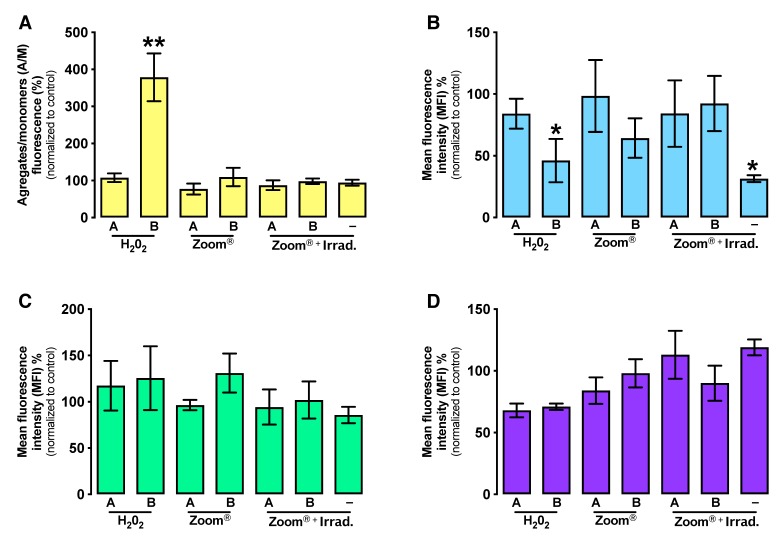
Oxidative stress analysis. (**A**) Mitochondrial membrane potential evaluation determined using the fluorescent probe JC-1. (**B**) Production of intracellular peroxides using DCFH2-DA. (**C**) Production of superoxide radical using DHE. (**D**) Expression of GSH using orange mercury. –: no compound administration. Statistical differences from the respective control are indicated by *, where * represents *p* < 0.05 and ** *p* < 0.01. The results express the average of 5 independent experiments ± standard error.

Due to its importance in cell metabolism and an important origin of ROS, mitochondrial membrane potential was evaluated, since it evaluates the membrane integrity. Figure 3A shows the ratio between monomers and aggregates in cell cultures after treatment. A marked increase in membrane potential in 0.01% HP application (*p* = 0.009) was observed.

### 3.4. Intracellular Peroxides and Superoxide Radical Production

The cells oxidative stress status was evaluated through ROS production and antioxidant defenses.

As seen in Figure 3B, peroxide production is statistically decreased after treatment with 0.01% HP (*p* = 0.047) and a higher decrease is seen at system light application (*p* = 0.014).

Despite some variations, superoxide radical production in different groups presented no statistical significance (Figure 3C).

### 3.5. GSH Expression

Glutathione represents an important defense against ROS, so its expression was evaluated and is presented in Figure 3D. In general, tested conditions cause a decrease in GSH activity, although with no statistical significance.

## 4. Discussion

Cell-based cytotoxic tests are a widely accepted methodology for testing dental materials’ cytotoxicity, with fibroblast cells being a convenient and reproducible model, as listed by ISO/EN10993-5 [13,14,24]. In addition to the well-known role of fibroblasts on collagen synthesis central to the extracellular matrix of the pulp tissue, these cells are fundamental for several other functions, including stem cell recruitment and differentiation, angiogenesis, and nerve sprouting and regeneration. These functions are regulated by growth factors produced by the fibroblasts (TGFβ1, FGF2, VEGF, and NGF) or released from components of the extracellular matrix (collagen I and IV, laminin, and fibronectin) [25]. Moreover, they play an essential role in dental pulp defense in cases of pulp infection or injury, mediating inflammatory response and stem-cell recruiting, and, together with odontoblasts, respond to oxidative stress through several mechanisms as an increase in syntheses of heme oxygenase-1 [26]. These reactions are catalyzed by specific enzymes, sensitive to ambient changes, like those originated by ROS in whitening treatments [27].

This way, fibroblasts play an essential role in pulp function and defense, thus justifying the interest in studying the cytotoxic effect of whitening products in these cells.

While the exact amount of whitening product that diffuses and reaches the pulp is variable and scantily characterized, values from 0.175 µg/mL to 40.4 µg/mL are stated for different concentrations and application times [27,28,29,30,31,32]. In this study, amounts between 34 mg (0.3%) and 0.113 µg (0.000001%) were tested, comprising all described concentrations, and an exposure time of 45 min was performed close to clinical use [31].

Results from MTT assay support that the tooth whitening product tested induces less oxidative stress than HP, although some inhibition of cell metabolic activity occurs in all groups, as none of the Zoom^®^ conditions induce irreversible modifications, and cell recovery is verified in the Zoom^®^ groups (G2 and G3), but not in the HP group (G1). These results also reinforce previous results that whitening agents present some cytotoxicity in a dose-dependent manner [33]. Furthermore, we were able to determine a time point for cell recovery of 24 h for Zoom^®^ and 48 h for Zoom^®^ + irradiation. Previous studies also reported cell recovery at 72 h for other whitening products, so we hypothesize that despite different brand compositions, the presence of agents in the composition of products is responsible for cell protection [10,34]. Although we did not examine the effect of individual ingredients in Zoom^®^ composition, present compounds as glycerin and some polymers make the product thicker, decreasing the release of ROS from the product, which can explain these results. Moreover, the presence of agents as potassium nitrate can possibly prevent some cytotoxicity in clinical use since a decrease in dental hypersensitivity associated with dental bleaching is observed with its use [35]. Regarding the referred studies, the evaluation time was only at 0 h and 72 h, so we performed an evaluation at more times to more precisely determine cell recovery in line with clinical experience, since tooth whitening sensitivity is more frequent at in-office treatments and usually lasts longer.

Photoactivation of Zoom^®^ raises medium-term cytotoxicity (cellular recovery at 48 h), which is probably due to an increase in ROS formation and acceleration of oxidation-reduction reactions by the system lamp [36]. Because an increase in temperature promoted by the system could increase the cytotoxicity, we deviated the light 30 cm from the test plates, eliminating heat as the cause of the increased cytotoxicity observed groups G3 and G4. The manufacturer indicates that the system lamp emits LED light (wavelength emissions between 400 and 505 nanometers); however, results in group G4 might indicate that more energy radiation is emitted, namely, high-energy visible light, with a cytotoxic effect per se, as previously described in vivo, so further research is needed on this topic [37]. Although the direct cytotoxic action of light can be verified in vitro, it is unlikely to occur in vivo due to the protective effects of the dentin and enamel layer, which prevent light radiation from reaching the cells within the pulp, and acts as an isolator to temperature increase [17].

Activation of the death pathways was investigated by flow cytometry, and MTT results were confirmed, supporting Zoom^®^ inducing less oxidative stress. An increase in apoptotic cells (IC_50_) and a decrease in live cells (0.01%) was seen in the HP group, but no differences were seen in the Zoom^®^ groups. This increase in apoptotic cells is seen after 45 min and is related to HP concentration. Previous studies support these results and reported that, for fibroblasts, apoptosis could be initiated soon as 1 h for HP concentrations between 0.8 and 1.6 mM [38]. Regarding cell death type, some authors mention a necrosis death type, which can be explained by different exposure protocols and products which originate different amounts of ROS formation and also by different cell types, since the sensitivity of cells responding to oxidative stress is cell-type-specific [11,33]. If relevant, a more detailed distinction between apoptosis and necrosis or even necroptosis type of death could be determined using other markers, like receptor-interacting protein (RIP) [38] or through differential gene expression patterns [39]. Since the capacity of HP to induce cell death is cell-specific, the same concentration can have a different impact on other cell types, like odontoblasts.

Exposure to HP is known to trigger apoptosis trough the mitochondrial pathway, and the loss of mitochondria membrane potential is observed [38]. In the present study, mitochondrial membrane potential evaluation reinforces that Zoom^®^ groups showed no differences regarding control, with only the 0.01% HP group presenting a very significant increase in M/A ratio, signaling marked oxidative stress. It is known that a higher mitochondrial membrane potential correlates with higher ROS production, which can justify the increase in apoptosis and decrease in life cells found in this group. Although previous studies verified an increase in PI-positive cells, which they associated with cell necrosis [40], in the present study, only the HP group presented an increase in cell membrane potential, which supports our findings of the leading cause of cell death being apoptosis.

In the sequence, we analyzed some ROS of importance, namely, peroxides and superoxide radical production. No significant increase was seen and a significant decrease in peroxides production was seen in the 0.01% HP (*p* = 0.047) and irradiation only (*p* = 0.014) groups. These results confirm the observed increased cytotoxicity and cell death by apoptosis, since peroxides production evaluation only accounts for live cells, being those apoptotic, and consequently with higher peroxides production excluded from the analysis. Cell damage in these groups can occur by changes in the cellular redox status and intracellular ROS production, but also by direct ROS action, like lipid peroxidation, protein or DNA damage [41,42]. This hypothesis agrees with the increase in cell death by apoptosis, since protein damages promote an increase in pro-apoptotic proteins and a decrease in anti-apoptotic proteins, with subsequent cell death by apoptosis. Furthermore, studies with antioxidants like naringin, ascorbic acid, or vitamin E, showed prevented cellular cytotoxicity from hydrogen peroxide, clearly demonstrating the involvement of ROS in cell damage in a direct way [26,41,42]. We also cannot exclude the production of other ROS, such as the hydroxyl radical or singlet oxygen, not evaluated in this study [43].

The oxidative defense analyzed, reduced glutathione, shows no statistical differences between groups, which can be explained by the short time of evaluation and because reduced GSH concentration is maintained at a constant concentration, even in oxidative stress conditions [37]. Moreover, other defenses may be activated instead of glutathione, such as catalase or superoxide dismutase [43].

The results of cell recovery observed in Zoom^®^ groups but not in the HP groups, support the oxidative stress protective role of other components in the composition of tooth whitening products. Although careful extrapolation, since this is an in vitro study, these results also back up the use of bleaching products to safely restore vital discolored teeth because its side effects are temporary and transient, allowing cell recovery. This study brings new information about whitening products safety and mechanism of action; however, some limitations can be stated. First, this study evaluates only one specific product, so we cannot assume all commercial whitening products present the same response due to different compositions. Of importance, bleaching products’ cytotoxic effect is a complex phenomenon involving the pulp complex and several cell types, with an inflammatory component besides the cell death, so more comprehensive studies are needed to elucidate all the mechanisms. The observed cellular death can only partially explain the side effects associated with dental bleaching procedures.

Furthermore, being an in vitro study, caution should be taken when extrapolating the results obtained to the clinical practice since in vivo factors as intratubular fluid pressure, antioxidants defenses, or extracellular matrix protect the pulp, reducing the cytotoxicity of the whitening products.

In the future, studies of products with different HP percentages could add more information about the HP threshold that can be safely used at clinical practice. Moreover, studies with other cell types, as odontoblasts, and analysis of other ROS and inflammatory molecules can complement the information obtained.

## 5. Conclusions

We can conclude that cell death caused by whitening agents is dose-dependent and due to ROS generation and oxidative stress, leading to cell death mainly by apoptosis. The light activation of whitening products increases the cytotoxicity, although there is a cellular recovery in time.

Our results show cell recovery in the Zoom^®^ (24 h) and Zoom^®^ + irradiation (48 h) groups, which did not occur for HP alone. Obviously, caution should be taken when extrapolating to the clinic; however, these results support that whitening products with moderate HP concentration have a temporary effect on cells, allowing a cellular recovery.

This study supports the use of bleaching products to safely restore vital discolored teeth because its side effects are temporary and transient, allowing cell recovery.

## Figures and Tables

**Table 1:** Half-maximal inhibitory concentration (IC_50_) of fibroblast cells after exposure to the three conditions.

Group	Evaluation Time after Exposure (h)	IC_50_ (%)	R^2^	Confidence Intervals (Lower–Upper)
**Hydrogen Peroxide**	0	1.43 × 10^−3^	0.94	9.24 × 10^−4^–2.22 × 10^−3^
	24	7.92 × 10^−4^	0.96	7.34 × 10^−4^–8.55 × 10^−4^
	48	5.62 × 10^−4^	0.99	5.11 × 10^−4^–6.18 × 10^−4^
	72	4.34 × 10^−4^	0.98	3.90 × 10^−4^–4.83 × 10^−4^
**Zoom** **^®^**	0	6.71 × 10^−4^	0.91	3.76 × 10^−4^–1.20 × 10^−3^
	24	6.61 × 10^−4^	0.95	3.68 × 10^−4^–1.19 × 10^−3^
	48	5.26 × 10^−3^	0.95	0–2.74 × 10^−2^
	72	1.16 × 10^−2^	0.96	9.02 × 10^−3^–1.49 × 10^−2^
**Zoom** **^®^** **+ irradiation**	0	1.18 × 10^−2^	0.88	7.76 × 10^−3^–1.80 × 10^−2^
	24	8.51 × 10^−3^	0.95	7.13 × 10^−3^–1.02 × 10^−2^
	48	8.05 × 10^−4^	0.97	6.74 × 10^−4^–9.61 × 10^−4^
	72	1.90 × 10^−3^	0.97	1.23 × 10^−3^–2.93 × 10^−3^

R^2^ represents the coefficient of determination of the fitted curves. This value represents the average of at least 6 independent experiments.
